# Angiopoietin-2 as a Prognostic Biomarker of Major Adverse Cardiovascular Events and All-Cause Mortality in Chronic Kidney Disease

**DOI:** 10.1371/journal.pone.0135181

**Published:** 2015-08-14

**Authors:** Yi-Chun Tsai, Chee-Siong Lee, Yi-Wen Chiu, Hung-Tien Kuo, Su-Chu Lee, Shang-Jyh Hwang, Mei-Chuan Kuo, Hung-Chun Chen

**Affiliations:** 1 Graduate Institute of Clinical Medicine, College of Medicine, Kaohsiung Medical University, Kaohsiung, Taiwan; 2 Division of General Medicine, Kaohsiung Medical University Hospital, Kaohsiung, Taiwan; 3 Division of Nephrology, Kaohsiung Medical University Hospital, Kaohsiung, Taiwan; 4 Division of Cardiology, Kaohsiung Medical University Hospital, Kaohsiung, Taiwan; 5 Faculty of Renal Care, Kaohsiung Medical University, Kaohsiung, Taiwan; 6 Institute of Population Sciences, National Health Research Institutes, Miaoli, Taiwan; University of Leicester, UNITED KINGDOM

## Abstract

**Background:**

Chronic kidney disease (CKD) patients have higher prevalence of major adverse cardiovascular events (MACE) and all-cause mortality. Endothelial damage and dysfunction have been regarded as early portents of MACE in CKD patients. Angiopoietin-2 (Ang-2) impairs endothelial function and promotes aberrant neovascularization. The aim of the study was to assess the relationship between circulating Ang-2 and MACE or all-cause mortality in a CKD cohort.

**Methods:**

A total of 621 pre-dialysis stage 3–5 CKD patients were enrolled from January 2006 to December 2011 and were followed up till October 2014. Plasma Ang-2 was measured in duplicate using commercial enzyme-linked immunosorbent assays (ELISA). Clinical outcomes included MACE or all-cause mortality

**Results:**

Of all patients, 122 (19.8%) reached MACE or all-cause mortality. Seventy-two had MACE, 79 died, and 29 had both MACE and all-cause mortality during the follow-up period of 41.5±28.3 months. Ang-2 quintile was divided at 1405.0, 1730.0, 2160.9, and 2829.9 pg/ml. The adjusted HR of MACE or all-cause mortality for every single higher log Ang-2 was 5.69 (95% CI: 2.00–16.20, P = 0.001). The adjusted HR of MACE or all-cause mortality was 2.48 (95% CI: 1.25–4.90) for patients of quintile 5 compared with those of quintile 1. A longitudinal association between MACE or all-cause mortality and stepwise increases in Ang-2 levels was found (P-trend = 0.008).

**Conclusions:**

Ang-2 is an independent predictor of MACE or all-cause mortality in CKD patients. Additional study is necessary in order to explore the mechanism of the association of Ang-2 with adverse outcomes in patients with CKD.

## Introduction

Chronic kidney disease (CKD) has been recognized as a worldwide health issue [1] and CKD patients have higher risk of developing major adverse cardiovascular events (MACE) and all-cause mortality compared to the general population [2, 3]. The pathophysiology and mechanisms of the increased incidence of MACE and mortality in CKD population remain complex. Traditional risk factors, such as age, diabetes, hypertension and hyperlipidemia are not powerful enough to predict survival or the development of MACE. Hence, non-traditional risk factors, including endothelial dysfunction have been regarded as important outcome predictors [4]. Endothelial damage and dysfunction are probably some early portents of MACE in CKD patients [5].

Angiopoietin, one of the endothelial growth factors, plays an important role in vascular development and remodelling [6]. Angiopoietin-1 (Ang-1), which binds to the Tie-2 receptor, springs signaling that stabilizes endothelial and vascular structure and promotes development and maturation of new vessels [7, 8]. Conversely, Ang-2 is released from Weibel-Palade bodies (WPB) by several stimuli and acts as a natural antagonist of Ang-1 through interfering with Ang-1-Tie-2 signaling. Ang-2 supports vessel regression in the absence of vascular endothelial growth factor (VEGF), but assists in endothelial cell migration and proliferation with VEGF [9]. Elevated Ang-2 may contribute to aberrant neovascularization and endothelial abnormalities.

Previous observational studies reported increased circulating Ang-2 in atherosclerotic vascular diseases, such as coronary artery disease [10], congestive heart disease [11] and peripheral artery disease [12]. Furthermore, the Anglo-Scandinavian Cardiac Outcomes Trial (ASCOT) study showed that circulating Ang-2 was predictive of cardiovascular disease in patients with hypertension [13]. Lorbeer et al. demonstrated a significant association of circulating Ang-2 with cardiovascular and all-cause mortality in the general population [14]. Additionally, Ang-2 is markedly elevated in patients with CKD either on dialysis or not [15]. Ang-2 has been regarded as a clinical indicator of early cardiovascular disease in children on dialysis [16]. Molnar et al. found that circulating Ang-2 could predict mortality in kidney transplant recipients as well [17]. David et al. reported an association of Ang-2 with all-cause mortality in a study population consisted of 43 CKD stage 4 and 85 dialysis patients [18]. For patients with CKD not on dialysis, the evidence of the association of Ang-2 with MACE or all-cause mortality is lacking, because of relatively small number of those patients. Our recent report showed a significant correlation between circulating Ang-2 and renal progression in CKD patients who were not on dialysis [19]. We further hypothesized that circulating Ang-2 could also have prognostic implications in CKD patients with increased risk for MACE and all-cause mortality. Hence, the aim of this study is to evaluate whether Ang-2 is associated with MACE or all-cause mortality in patients with CKD stages 3–5.

## Materials and Methods

### Study Participants

As reported previously [19], we enrolled 621 patients with CKD stages 3–5 at a hospital in southern Taiwan from January 2006 to December 2011. CKD was staged according to K/DOQI definitions and the estimated glomerular filtration rate (eGFR) was calculated using the equation of the 4-variable Modification of Diet in Renal Disease (MDRD) Study (CKD stage 3, eGFR: 30~59 ml/min/1.73m^2^; CKD stage 4, eGFR: 15~29 ml/min/1.73m^2^; CKD stage 5, eGFR<15 ml/min/1.73m^2^) [20]. We excluded 6 patients reaching cardiovascular event or all-cause mortality within 30days after enrollment to avoid the impact of underlying diseases on clinical outcome. The study protocol was approved by the Institutional Review Board of the Kaohsiung Medical University Hospital (KMUH-IRB-990198). Participants had written informed consents, and all clinical investigations were conducted according to the principles expressed in the Declaration of Helsinki.

### Measurement of Circulating Angiopoietin-2

Blood samples were collected at enrollment. Plasma Ang-2 was measured using commercial enzyme-linked immunosorbent assays (ELISA, R&D Systems Inc, Minneapolis, MN) based on the instructions of the manufacturer. The sensitivity of Ang-2 assay was 1.20 pg/ml. Intraassay and interassay coefficients of variation of Ang-2 were 1.8% and 1.2% respectively. All assays were performed in duplicate by investigators blinded to patient characteristics and clinical outcomes.

### Clinical Measurements

All demographic and clinical information including age, gender, cigarette smoking, alcohol consumption, and co-morbidity were obtained from medical records and patient interviews at enrollment. Blood sample was collected for the biochemistry study after 12 hours fasting. Dipstick test was used for measuring the severity of proteinuria, and graded as negative, trace, 1+, 2+, 3+, or 4+. Data of patient medications including β-blocker, calcium channel blocker, angiotensin converting enzyme inhibitor (ACEI), and angiotensin II receptor blocker (ARB) before and after enrollment was obtained from medical records. Patients were classified as hypertensive defined as those with a history of hypertension, current use of antihypertensive drugs, or where the measurement of blood pressure was more than 140/90mmHg. Diabetes was defined by history and blood glucose values using the American Diabetes Association criteria, oral hypoglycemia agent or insulin use. Heart disease was defined as having a history of congestive heart failure, acute or chronic ischemic heart disease, or myocardial infarction. Cerebrovascular disease was defined as having a history of brain infarction or hemorrhage.

### Clinical Outcomes

Clinical outcomes included MACE or all-cause mortality. MACE was defined as new onset of acute myocardial infarction, acute hemorrhagic or ischemic stroke, and hospitalization related to acute phase of congestive heart failure. Patients were contacted at outpatient clinics at 3-month intervals to ascertain the clinical status. At least three telephone calls were made to the patient at his/her last known telephone number if patients had irregular follow-up. The information obtained by direct contact with patients and families was further supplemented by reviewing medical records and screening the data bank of the National Mortality File. Patients were censored at the last contact, initial commencing dialysis, or the end of observation in October 2014. We excluded cardiovascular events or death after commencing dialysis.

### Statistical Analysis

Statistical results of baseline characteristics of all patients were stratified by quintiles of Ang-2, cut at 1405.0, 1730.0, 2160.9, and 2829.9 pg/ml. Continuous variables were expressed as mean±SD or median (25^th^, 75^th^ percentile) as appropriate, and categorical variables were expressed as percentages. Skewed distribution of continuous variables was log-transformed to approximate normal distribution. The significance of differences in continuous variables between groups was tested using one-way analysis of variance (ANOVA) or the Kruskal-Wallis H test as appropriate. Post hoc multiple comparisons with Bonferroni method was used to compare the dissimilarity among the two groups. The difference in the distribution of categorical variables was tested using the Chi-square test. Time-to-event survival analysis by Kaplan-Meier survival curve was used to test Ang-2 as a predictor of the risk of MACE or all-cause mortality. The association between Ang-2 and MACE or all-cause mortality was assessed by a modified stepwise procedure in three modeling steps. The first model consisted of age and sex. The second model consisted of adding smoking history, diabetes mellitus, heart disease, and medication including ACEI/ARB or β-blocker. The third step was adding renal function status and biochemical factors, including body mass index, eGFR, urine protein, serum hematocrit, uric acid and cholesterol, and log serum albumin and phosphate levels. Statistical analyses were conducted using SPSS 18.0 for Windows (SPSS Inc., Chicago, Illinois). Statistical significance was set at a two-sided p-value of less than 0.05.

## Results

### Characteristics of the Entire Cohort

Table 1 shows the baseline demographic and clinical characteristics stratified by quintiles of Ang-2. The mean eGFR of 615 CKD patients was 21.8 ml/min/1.73m^2^ (145 in stage 3, 242 in stage 4, 228 in stage 5). The mean age was 65.3±12.6 years and 55.2% were male. 38.5% were diabetic mellitus, and 85.6% were hypertensive. Pre-existing and documented heart disease and cerebral vascular disease were noted in 17.7% and 8.8% of the patients respectively. Stepwise decreases in eGFR, serum hematocrit, albumin, and total calcium levels, and stepwise increases in proportion of β-blocker, serum blood urea nitrogen, phosphate and high-sensitivity C-reactive protein (hsCRP) levels, and urine protein corresponded to the advancement from quintile 1 to quintile 5.

**Table 1:** The clinical characteristics of study subjects stratified by angiopoietin-2 quintile.

		Angiopoietin-2^a^					
	Entire Cohort N = 615	Quintile 1 N = 124	Quintile 2 N = 124	Quintile 3 N = 120	Quintile 4 N = 124	Quintile 5 N = 123	P-trend
Demographics							
Age (year)	65.3±12.6	61.2±13.7^#^ ^†^	65.7±11.2*	68.4±12.2*	65.2±11.8	66.2±12.9*	<0.001
Sex (male, %)	55.2	64.5	58.1	55.4	48.4	49.6	0.07
Smoke (%)	19.6	24.2	13.7	16.7	20.7	22.8	0.21
Alcohol (%)	8.0	13.7	9.7	5.0	5.0	6.5	0.06
Cardiovascular disease (%)	17.7	17.7	14.5	14.0	22.6	19.5	0.38
Cerebral vascular disease (%)	8.8	8.1	7.3	13.2	8.9	6.5	0.36
Hypertension (%)	85.6	89.5	87.9	82.6	83.1	84.6	0.44
Diabetes mellitus (%)	38.5	30.6	37.9	36.4	39.5	48.0	0.08
Hyperlipidemia (%)	43.8	41.1	50.0	47.9	37.9	42.3	0.27
CKD cause							
Chronic glomerulonephritis (%)	37.5	37.1	37.1	36.4	37.1	39.8	0.82
Diabetic nephropathy (%)	29.1	25.0	29.8	29.8	27.4	33.3	
Others^b^ (%)	33.4	37.9	33.1	33.9	35.5	26.8	
CKD stage 3 (%)	23.5	30.6	25.0	24.0	19.4	18.7	0.01
4 (%)	39.4	41.9	42.7	42.1	40.3	30.1	
5 (%)	37.1	27.5	32.3	33.9	40.3	51.2	
Medications							
Calcium channel blocker (%)	54.7	52.4	55.6	57.0	46.8	61.8	0.19
β-blocker (%)	23.7	16.9	16.1	25.6	29.8	30.1	0.01
ACEI/ARB (%)	56.8	59.7	61.3	46.3	59.7	56.9	0.10
Statin (%)	27.3	25.0	28.2	29.8	22.6	30.9	0.55
Laboratory parameters							
Blood urea nitrogen (mg/dl)	41.1(30.0,60.0)	33.3(26.0,50.8)^&^	39.5(29.9,59.0)	37.4(28.2,52.2)^&^	45.9(33.3,62.8)* ^†^	50.1(35.5,66.3)* ^†^	<0.001
eGFR (ml/min/1.73m^2^)	21.8±12.6	24.9±13.9^&^	23.2±12.4	22.2±11.9	19.8±11.8*	18.8±12.2*	0.001
Creatinine	3.8±2.3	3.5±2.3	3.6±2.3	3.5±1.9	4.1±2.4	4.2±2.3	0.04
Fasting sugar (g/dl)	101(92,117)	100(93,111)	99(92,116)	100(92,120)	101(92,124)* ^†^	103(93,126)*	0.60
Glycated hemoglobin (%)	5.8(5.5,6.7)	5.7(5.5,6.1)	5.9(5.6,6.6)	5.8(5.4,6.8)	5.8(5.4,6.4)* ^†^	6.1(5.5,7.3)* ^†^	0.06
Hematocrit (%)	33.1±6.8	35.8±8.4^#^ ^&^	33.3±6.1*	33.4±6.7	31.6± 5.9*	31.2±5.7*	<0.001
Albumin (g/dl)	4.1(3.9,4.3)	4.2(4.0,4.4)^&^	4.1(4.0,4.3)	4.1(3.8,4.3)	4.0(3.7,4.2)*	4.0(3.7,4.2)* ^#^	<0.001
Calcium (mg/dl)	8.9±0.6	9.1±0.5^&^	9.0±0.6	8.9±0.6	8.8±0.6*	8.8±0.8* ^#^	<0.001
Phosphate (mg/dl)	4.1(3.6,4.7)	4.0(3.5,4.5)	4.0(3.6,4.7)	4.1(3.7,4.5)	4.2(3.6,4.9)	4.2(3.8,5.1)	0.03
Uric acid (mg/dl)	7.6±1.8	7.4±1.8	7.6±1.8	7.6±1.9	7.5±1.6	7.8±2.0	0.39
Cholesterol (mg/dl)	188±45	191±44	192±48	188±39	181±44	188±47	0.34
Triglyceride (mg/dl)	115(78,173)	116(78,181)	104(76,159)	139(78,202)	126(79,181)	124(74,177)	0.09
hsCRP (mg/L)	1.6(0.6,4.2)	1.5(0.7,4.2)	1.2(0.4,2.8)	1.5(0.7,3.6)	1.7(0.6,4.2)	2.1(0.6,6.4)* ^#^ ^†^	0.03
Parathyroid hormone (pg/ml)	72(37,157)	61(40,158)	61(35,120)	68(39,130)	87(39,211)	103(33,170)	0.38
Urine protein^c^							0.007
0 (%)	13.0	20.8	18.7	8.1	11.5	6.3	
1+ (%)	35.6	41.6	33.0	39.5	32.3	32.1	
2+ (%)	29.2	22.8	28.6	34.9	32.3	28.6	
3+ or more (%)	22.2	14.9	19.8	17.4	24.0	33.0	

Data are expressed as number (percentage) for categorical variables and mean±SD or median (25^th^, 75^th^ percentile) for continuous variables, as appropriate.

*P < 0.05 compared with quintile 1

^#^ *P* < 0.05 compared with quintile 2

^†^ *P* < 0.05 compared with quintile 3

^&^ *P* < 0.05 compared with quintile 4.

^a^Ang-2 quintiles cut at 1405.0, 1730.0, 2160.9, and 2829.9pg/ml

^b^Include tubular interstitial disease, obstructive nephrology, hypertensive nephrology and unknown causes.

^c^Urine protein was measured by dipstick test, urine protein 0 as no detectable proteinuria.

### Ang-2, All-Cause Mortality and Cardiovascular Events

Over a mean follow-up period of 41.5±28.3 months, 122 patients (20.2%) reached MACE or all-cause mortality (Table 2). Seventy-two had MACE, 79 died, and 29 had both MACE and mortality during the follow-up period. A stepwise increase in the proportion of MACE or all-cause mortality from quintile 1 to quintile 5 was found (P-trend = 0.03). Sepsis accounts for 38% of deaths, malignancy for 22%, MACE for 14% and the rest were a mixture of refusing dialysis and other causes. The causes of MACE were events of 24 acute myocardial infarction, 16 acute hemorrhagic or ischemic stroke, and 32 hospitalization due to congestive heart failure. There were no significant differences in the causes of death and cardiovascular events between the 5 groups. Twenty-five (4.1%) patients were lost to follow-up (the mean follow-up period: 21.2±12.9 months), and there was no significant differences in the proportion of loss follow-up and clinical characteristics from quintile 1 to quintile 5.

**Table 2:** Major adverse cardiovascular events (MACE) and all-cause mortality of cohort.

		Angiopoietin-2^a^					
	Entire Cohort N = 615	Quintile 1 N = 124	Quintile 2 N = 124	Quintile 3 N = 120	Quintile 4 N = 124	Quintile 5 N = 123	P-trend
Follow-up time (month)	41.5±28.3	49.2±29.9	40.3±27.4	41.2±27.4	41.8±28.6	34.9±26.2*	0.003
Composite outcomes^b^, n(%)	122(19.8)	16(12.9)	20(16.1)	24(20.0)	27(21.8)	35(28.5)	0.02
Major adverse cardiovascular events, n(%)	72(11.7)	11(8.9)	11(8.9)	11(9.2)	16(12.9)	23(18.7)	0.07
Death, n(%)	79(12.8)	9(7.3)	13(10.5)	17(14.2)	18(14.5)	22(17.9)	0.11
Sepsis, n(%)	30(4.9)	5(4.0)	4(3.2)	8(6.6)	7(5.6)	6(4.9)	0.41
Malignancy, n(%)	17(2.8)	1(0.8)	2(1.6)	1(0.8)	6(4.8)	7(5.7)	
Cardiovascular cause, n(%)	11(1.8)	2(1.6)	2(1.6)	2(1.7)	2(1.6)	3(2.4)	
Refuse dialysis, n(%)	6(0.9)	0(0)	0(0)	4(3.3)	0(0)	2(1.6)	
Others, n(%)	8(1.3)	0(0)	3(2.4)	1(0.8)	2(1.6)	2(1.6)	
Natural death at home, n(%)	7(1.1)	1(0.8)	2(1.6)	1(0.8)	1(0.6)	2(1.6)	

*P < 0.05 compared with quintile 1

^#^ *P* < 0.05 compared with quintile 2

^†^ *P* < 0.05 compared with quintile 3

^&^ *P* < 0.05 compared with quintile 4.

^a^Ang-2 quintiles cut at 1405.0, 1730.0, 2160.9, and 2829.9pg/ml

^b^Composite outcomes including major adverse cardiovascular events or all-cause mortality.

**Fig 1:**
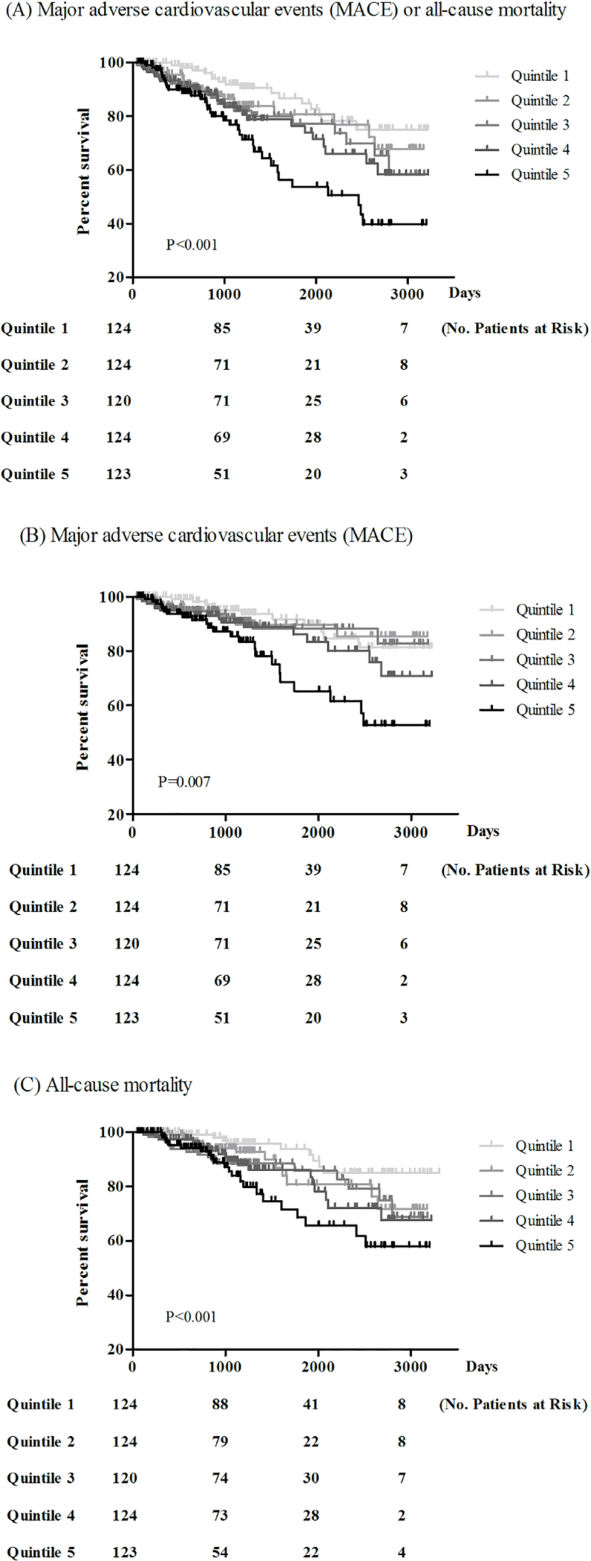
The cumulative probability of (A) major adverse cardiovascular events (MACE) or all-cause mortality (B) major adverse cardiovascular events (MACE) (C) all-cause mortality according to Angiopoietin-2 quintiles.

Kaplan-Meier survival curve showed a significant correlation between MACE or all-cause mortality and quintiles of Ang-2 (Fig 1A–1C). This significant result was tested either by treating Ang-2 as continuous and category variable in the univariate cox proportional model and multivariate stepwise model analysis. The unadjusted and adjusted (model 3) hazard ratio (HR) of MACE or all-cause mortality for every single higher log Ang-2 was 8.41 (95% confidence interval (CI): 3.38–20.91, P<0.001) and 5.78 (95% CI: 2.02–16.50, P = 0.001) respectively. The unadjusted and adjusted (model 3) HR of MACE or all-cause mortality was 3.23 (95% CI: 1.78–5.84) and 2.49 (95% CI: 1.26–4.93) for patients of quintile 5 compared with those of quintile 1. There was a longitudinal association between MACE or all-cause mortality and stepwise increases in Ang-2 levels (P-trend = 0.008, Table 3). We performed subgroup analysis and Ang-2 level was positively and significantly correlated with MACE or all-cause mortality in patients with hsCRP level less than 1 mg/dl (HR for every log Ang-2: 10.97, 95%CI: 1.14–105.91) and in those with hsCRP level of 1 or more (HR: 4.58, 95%CI: 1.40–14.98) after adjusting age, sex, cigarette smoking, medication and co-morbidities.

**Table 3:** The adjusted risks for major adverse cardiovascular events (MACE) and all-cause mortality according to Angiopoietin-2 level.

	Unadjusted	Model 1	Model 2	Model 3
Angiopoietin-2	HR(95% CI)	HR(95% CI)	HR(95% CI)	HR(95% CI)
***Major adverse cardiovascular events (MACE) or all-cause mortality***				
Continuous	8.41(3.38–20.91)	6.89(2.66–17.85)	6.10 (2.33–15.93)	5.78 (2.02–16.50)
Quintile 1	1(Reference)	1(Reference)	1(Reference)	1(Reference)
Quintile 2	1.51 (0.78–2.91)	1.32 (0.68–2.55)	1.36 (0.70–2.65)	1.33 (0.63–2.81)
Quintile 3	1.83 (0.97–3.45)	1.40 (0.74–2.65)	1.36 (0.71–2.60)	1.32 (0.64–2.70)
Quintile 4	1.99 (1.07–3.69)	1.69 (0.91–3.16)	1.58 (0.84–2.96)	1.51 (0.75–3.04)
Quintile 5	3.23 (1.78–5.84)	2.70 (1.48–4.93)	2.59 (1.41–4.75)	2.49 (1.26–4.93)
P-trend	<0.001	0.001	0.002	0.008
***Major adverse cardiovascular events (MACE)***				
Continuous	5.68(1.76–18.36)	5.36(1.61–17.92)	3.87(1.17–12.85)	4.01(1.08–14.84)
Quintile 1	1(Reference)	1(Reference)	1(Reference)	1(Reference)
Quintile 2	1.21 (0.53–2.80)	1.13 (0.49–2.62)	1.18 (0.50–2.76)	1.23 (0.47–3.27)
Quintile 3	1.22 (0.53–2.81)	1.06 (0.45–2.47)	0.99 (0.41–2.37)	1.19 (0.45–3.09)
Quintile 4	1.71 (0.79–3.68)	1.61 (0.74–3.49)	1.53 (0.70–3.32)	1.74 (0.72–4.22)
Quintile 5	3.03 (1.47–6.22)	2.85 (1.37–5.92)	2.45 (1.17–5.11)	2.58 (1.09–6.10)
P-trend	0.001	0.002	0.009	0.01
***All-cause mortality***				
Continuous	11.74(3.80–36.21)	7.86(2.38–26.01)	8.87(2.57–30.68)	10.28(2.62–40.38)
Quintile 1	1(Reference)	1(Reference)	1(Reference)	1(Reference)
Quintile 2	1.76 (0.75–4.13)	1.45 (0.62–3.42)	1.56 (0.66–3.70)	2.38(0.86–6.55)
Quintile 3	2.24 (1.00–5.03)	1.52 (0.67–3.43)	1.41 (0.61–3.27)	1.85 (0.69–4.94)
Quintile 4	2.41 (1.08–5.36)	1.78 (0.79–3.99)	1.70 (0.75–3.83)	2.11 (0.82–5.46)
Quintile 5	3.64 (1.67–7.92)	2.63 (1.20–5.81)	2.84 (1.28–6.30)	3.75 (1.45–9.71)
P-trend	0.001	0.01	0.01	0.01

Ang-2 quintiles cut at 1405.0, 1730.0, 2160.9, and 2829.9pg/ml

Unadjusted model is as no adjustment of other covariates

Multivariate model 1 is adjusted for age and sex

Multivariate model 2 comprises model 1 as well as smoke, diabetes mellitus, heart disease, β-blocker or angiotensin converting enzyme inhibitors/angiotensin II receptor blockers use

Multivariate model 3 comprises model 2 as well as body mass index, estimated glomerular filtration rate, urine protein, log serum albumin, log serum phosphate, hematocrit, serum uric acid and cholesterol levels.

The unadjusted and adjusted (model 3) HR of MACE for every single higher log Ang-2 was 5.68 (95% CI: 1.76–18.36, P = 0.004) and 4.01 (95% CI: 1.08–14.84, P = 0.03) respectively. The unadjusted and adjusted (model 3) HR of MACE was 3.03 (95% CI: 1.47–6.22) and 2.58 (95% CI: 1.09–6.10) for patients of quintile 5 compared with those of quintile 1. There was a longitudinal association between MACE and stepwise increases in Ang-2 levels (P-trend = 0.01).

The unadjusted and adjusted (model 3) HR of all-cause mortality for every single higher log Ang-2 was 11.74 (95% CI: 3.80–36.21, P<0.001) and 10.28 (95% CI: 2.62–40.38, P = 0.001) respectively. The unadjusted and adjusted (model 3) HR of all-cause mortality was 3.64 (95% CI: 1.67–7.92) and 3.75 (95% CI: 1.45–9.71) for patients of quintile 5 compared with those of quintile 1. There was a longitudinal association between all-cause mortality and stepwise increases in Ang-2 levels (P-trend = 0.01).

We also analyzed the association between Ang-2 and cancer mortality and the HR for every log Ang-2 increment was 74.20 (95%CI: 3.82–1443.23, P = 0.004) in adjusted analysis of model 3. There was a longitudinal association between cancer mortality and stepwise increases in Ang-2 levels (P-trend = 0.006, S1 Table).

## Discussion

This study shows that elevated circulating Ang-2 levels were associated with MACE or all-cause mortality in patients with stages 3–5 CKD over an observation period of 3 years. Patients with quintile 5 of Ang-2 had 2-fold increases in risk for MACE or all-cause mortality after adjustment of associated risk factors. Ang-2 is an independent predictor of MACE or all-cause mortality in CKD.

Our study found a significant relationship between Ang-2 and all-cause mortality, but the major cause of death was sepsis and it was very different with those in previous reports. In the study of David et al., cardiovascular events comprised the most proportion of deaths (47%) in patients with CKD stage 4 and maintenance dialysis [18]. The study of Health in Pomerania (SHIP) reported the cardiovascular causes and cancer were the major cause of death in Germany general population [14]. There are some explanations for this dissimilarity. Probably more Asian CKD patients progress to commencing dialysis rather than dying of cardiovascular events compared with those in western countries [21, 22]. Additionally, sepsis is a state of systemic vascular inflammation and endothelial dysfunction. WPB exocytosis-mediated Ang-2 is an important factor participating in inflammation and sepsis. Aside from Ang-2, WPB contains many other molecules, such as endothelin, interleukin-8, and von Willebrand factor [23], which lead to uncontrolled activation of the inflammatory and coagulation pathways. Endothelial cells probably communicate with inflammatory cells through the Ang/Tie system [23]. Thus, Ang-2 has been regarded as a useful marker of organ failure and mortality in sepsis [24]. Besides, malignancy is the second cause of mortality in our study, as well as the results in SHIP [14]. The Ang/Tie system has presented an attractive opportunity of cancer treatment [25]. Overexpression of Ang-2 may promote tumorigenesis and has been correlated with poor prognosis in various cancers. We analyzed the association between Ang-2 and cancer mortality and the results revealed a significant correlation after adjusting traditional risk factors, such as age, sex, cigarette smoking, co-morbidities, body mass index, renal function and associated biochemistry parameters. Elevated circulating Ang-2 level was also an independent risk factor for cancer mortality in CKD population.

Vascular and endothelial dysfunction is one of the major characteristics of CVD. The Ang-Tie system has been regarded as a key regulator of vascular maintenance and quiescent endothelial cell homeostasis. Ang-2 level is an indicator of the accelerated development of atherosclerotic burden [12]. Overexpression of Ang-2 augmented endothelial apoptosis and played a critical role in the progression of myocardial fibrosis in animal model [26]. From clinical views, Shroff et al. indicated the association of high Ang-2 levels with the increase in carotid intima media thickness in children on dialysis [16]. Additionally, Chang enrolled 416 CKD patients to investigate the potential role of arterial stiffness and found a significant correlation between Ang-2 and pulse wave velocity [27]. Lorbeer et al. conducted a cross-sectional study and showed elevated Ang-2 levels were associated with impaired left ventricular systolic function [28]. These findings indicated an association of Ang-2 with impaired vascular and cardiac function. Nevertheless, the relationship between Ang-2 and MACE is still not well-explored. Our results demonstrated that elevated circulating level was significantly associated with MACE in CKD population after adjusting established cardiovascular risk factors, such as age, sex, co-morbidity, renal function and associated biochemistry parameters. Ang-2 is a potential predictor for patients at an increased risk for MACE. Further study is needed to evaluate the precise mechanisms mediating this increased cardiovascular risk by Ang-2.

Inflammation also plays a key role in the atherosclerosis processes, further promoting to cardiovascular morbidity or mortality. Elevated Ang-2 induces inflammatory gene expression [29], and sensitizes the endothelium to inflammatory response [30]. Ang-2 increases neutrophil accumulation in tissues [31], and promotes neutrophil margination and stimulates neutrophil adherence [31, 32]. Additionally, Ang-2 can act as a modulator of the inflammatory response by promoting vascular leakage [31]. Accumulating evidence shows a positive association between Ang-2 and diseases with systemic inflammation including diabetes, CKD and cardiovascular disease [10, 27, 33]. To abate the effect of the interaction between Ang-2 and inflammation on adverse clinical outcomes, we adjusted hsCRP in multivariate analysis and it did not alter the significance. Furthermore, we performed subgroup analysis and the results still revealed a positive and significant association between Ang-2 level and MACE or all-cause mortality in patients with hsCRP level less than 1 mg/dl and those with hsCRP level of 1 or more after adjusting age, sex, cigarette smoking and co-morbidities. Thus, Ang-2 is an important risk factor for MACE and all-cause mortality independent of inflammation in CKD patients.

This study has some limitations that must be considered. The major uncertainty is whether circulating Ang-2 has biologically active effect in CKD patients. The biological implication of Ang-2 changes in the range observed in our patients is still unclear. Besides, Ang-2 and laboratory parameters were measured once at enrollment. The effect of the time-varying Ang-2 levels and laboratory parameters might be underestimated. Finally, we did not measure circulating Ang-1 and VEGF levels in this study. From a pathophysiologic perspective, it might be helpful for us to further clarify the possible linking effects of these markers.

In conclusion, our study demonstrated that elevated circulating Ang-2 is associated with increased risks for MACE or all-cause mortality in stages 3–5 CKD patients. Future studies will be necessary to evaluate the pathogenic role of Ang-2 in MACE or all-cause mortality, and moreover, to establish the beneficial clinical outcome through targeting Ang-2.

## Supporting Information

S1 Table The adjusted risks for cancer mortality according to Angiopoietin-2 level. (DOC)
