# Supplementary material for: Angiopoietin-2 as a Prognostic Biomarker of Major Adverse Cardiovascular Events and All-Cause Mortality in Chronic Kidney Disease
Source: PLoS One. 2015 Aug 14;10(8):e0135181. doi: 10.1371/journal.pone.0135181 (PMC4537136; doi:10.1371/journal.pone.0135181)
Supplement: S1 Table — (DOC) [file pone.0135181.s001.doc]

S1 Table. The adjusted risks for cancer mortality according to Angiopoietin-2 level

|  | Unadjusted | Model 1 | Model 2 | Model 3 |
| --- | --- | --- | --- | --- |
| Angiopoietin-2 | HR(95% CI) | HR(95% CI) | HR(95% CI) | HR(95% CI) |
| Continuous | 57.08 (5.16-631.22) | 45.00 (3.63-557.69) | 86.50 (6.19-1209.66) | 74.2 (3.82-1443.23) |
| Quintile 1 | 1(Reference) | 1(Reference) | 1(Reference) | 1(Reference) |
| Quintile 2 | 2.48 (0.22-27.38) | 2.08 (0.19-23.03) | 2.30 (0.21-25.86) | 1.41 (0.07-28.21) |
| Quintile 3 | 1.23 (0.08-19.60) | 0.84 (0.05-13.49) | 0.71 (0.04-11.56) | 0.70 (0.04-13.33) |
| Quintile 4 | 7.33 (0.88-60.96) | 5.46 (0.65-46.01) | 5.88 (0.69-50.17) | 8.61 (0.81-91.99) |
| Quintile 5 | 10.49 (1.29-85.46) | 7.86 (0.94-65.55) | 9.85 (1.17-83.09) | 8.71 (0.82-92.19) |
| P-trend | 0.003 | 0.007 | 0.003 | 0.006 |

Ang-2 quintiles cut at 1405.0, 1730.0, 2160.9, and 2829.9pg/ml

Unadjusted model is as no adjustment of other covariates

Multivariate model 1 is adjusted for age and sex

Multivariate model 2 comprises model 1 as well as smoke, diabetes mellitus, heart disease, β-blocker or angiotensin converting enzyme inhibitors/angiotensin II receptor blockers use

Multivariate model 3 comprises model 2 as well as body mass index, estimated glomerular filtration rate, urine protein, log serum albumin, log serum phosphate, hematocrit, serum uric acid and cholesterol levels
